# Supplementary material for: The Construction and Exploration of a Comprehensive MicroRNA Centered Regulatory Network in Foxtail Millet (Setaria italica L.)
Source: Front Plant Sci. 2022 May 6;13:848474. doi: 10.3389/fpls.2022.848474 (PMC9121102; doi:10.3389/fpls.2022.848474)
Supplement: Supplementary file 5 [file Data_Sheet_5.zip › Sit-miR399a_Seita.9G131900.1_313_TPlot.pdf]

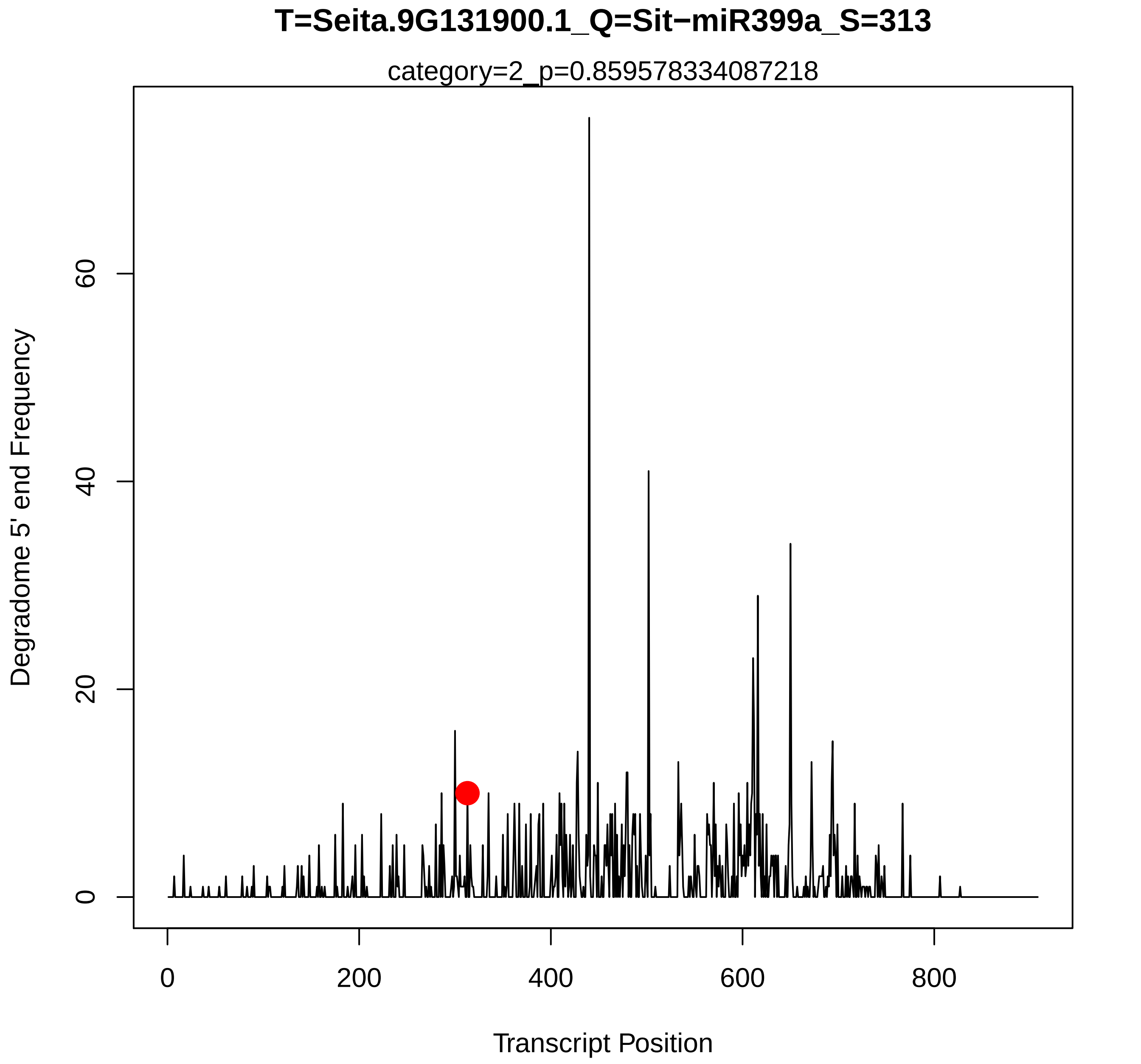

T=Seita.9G131900.1_Q=Sit−miR399a_S=313
category=2_p=0.859578334087218
Degradome 5' end Frequency
0
20
40
60
0
200
400
600
800
Transcript Position
